# Managing of post-traumatic knee arthritis by total knee arthroplasty: case series of 15 patients and literature review

**DOI:** 10.1186/s13018-019-1180-3

**Published:** 2019-05-31

**Authors:** Bahaa Ali Kornah, Hesham Mohamed Safwat, Said K. Abdel-hameed, Mohamed Abdel-AAl, Mohamed Abdelaziz, Mohamed I. Abuelesoud, Nagy Saleem

**Affiliations:** 1 Faculty of Medicine for Boys, Al-Azhar University, Cairo, Egypt; 2 Faculty of Medicine for Girls, Al-Azhar University, Cairo, Egypt; 3 Ministry of Health, Manshiet el Bakry Hospital, 61 Taha Heussein Street, Nozha, Heliopolis, Cairo, Egypt

**Keywords:** Total knee arthroplasty, Unstable arthritic knee, Post-traumatic arthritis, Bone defects

## Abstract

**Purpose:**

Post-traumatic arthritis is one of the leading causes of joint disability. This study aims at outlining outcomes of total knee arthroplasty in post-traumatic arthritis and technical difficulty and reviewing literature regarding this issue

**Patients and methods:**

We analyzed the outcome of total knee arthroplasty following post-traumatic arthritis in 15 patients with unilateral involvement. Ten had stable arthritic knees treated with posterior stabilized (PS) prosthesis, while five with unstable arthritic knees treated as follows: three with ligamentous instability managed by constrained condylar prosthesis and two with osseous deficiency, metal augmentation used together with stemmed constrained condylar prosthesis (CCK). Average follow-up 6 years, mean age 49.8 years at time of arthroplasty. Patient outcomes were evaluated on the basis of Knee Society score.

**Results:**

Mean clinical knee society scores (CKSS) at latest follow-up improved from 43.6 ± 11.66 points to 77.3± points postoperatively while mean functional knee society score (FKSS) improved from 40. ± 6.3 to 76.6 ± 84 postoperatively. Patients with stable knees had a higher mean values, both clinical and functional KSS, while unstable knees were poorer. Complications occurred in three cases, one with wound dehiscence with prolonged drainage treated by antibiotics and daily dressings until the wound closed completely, one was complicated by infection and improved by serial debridement, and the third case had aseptic loosening which required revision surgery.

**Conclusion:**

Total knee arthroplasty for post-traumatic arthritis decreases pain and improves knee function. However; the procedure is not as simple as primary arthroplasty as it is technically demanding and requires adequate planning.

## Introduction

Post-traumatic arthritis cases are considered types of osteoarthritis. However, cartilage degeneration that occurs in post-traumatic arthritis results from sudden injury and not gradual wear and tear as in case for osteoarthritis. Knee joint arthritis can occur due to many causes [1]. Trauma is a common predisposing factor for knee OA. This trauma extends from internal derangement of knee to intra-articular fractures and fracture dislocation [5].

The injury could be from sports, motor vehicle accident, fall, or any other source of physical trauma. Such injuries can damage the ligaments, cartilage, and/or the bone, changing the mechanics of the joint [4]. Post-traumatic knee OA accounts for 12% of all knee OA [13] and it always occurs in young patients which will affect the activity of daily living. A combination of factors most likely contributes to the development of post-traumatic arthritis (PTA) following injury to the knee. First, mechanical imbalance may be due to ligamentous laxity, meniscal tears, and malalignment [2]. Second, the release of pro-inflammatory cytokines into local tissue leads to imperfect remodeling of the cartilage. Lastly, nonunions and malunions following fractures may lead to PTA [8].

The treatment of post-traumatic knee OA varies from a combination of nonpharmacological and pharmacological modalities to operative interventions. Activity modification, anti-inflammatory medications, ambulatory assist devices, and physical therapy are the mainstay of nonoperative treatment [6].

With failure of conservative treatment, surgical options become an alternative tool ranging from arthroscopic debridement to arthrodesis [6]. Total knee arthroplasty (TKA) is an option for the treatment of end-stage post-traumatic knee OA [4]. It is not so simple but often more technically challenging due to previous surgeries and scarring [15], problems related to secondary deformity, poor bone quality, bone loss, and ligament incompetence, and it is always associated with higher complication rate. In addition, it accounts for more consumption of hospital resources and incurs a higher cost [14].

Our study aims at outlining the outcomes of total knee arthroplasty in post-traumatic arthritis as well as the technical difficulties of the procedure.

## Patients and methods

Between 2006 and 2014, 15 patients (12 males, 3 females) with unilateral post-traumatic knee OA were enrolled in this study. The study was initiated after receiving approval from the institutional ethics committee for research in accordance with the ethical standards laid down in the 1964 declaration of Helsinki and its later amendments. Also, a written consent had been obtained from the patient for participating in the study. All review authors independently performed study selection. Two authors independently assessed the included study and performed data extraction. Four cases had intra-articular distal femoral fractures and 11 had tibial plateau fractures. The mean age is 49.8 years (range, 38 to 65 years). Causative injuries were fall from height (nine patients), road traffic accidents (four patients), and in one case, drop of heavy box over the knee while working and one case due to heavy sporting. The average duration from trauma to presentation to our clinic was 19.5 months (range 11 to 36 months). Inclusion criteria involved patients below 50 years with severe pain and disability due to intra-articular fractures around the knee and exclusion criteria involved patients above 50 years, skeletally immature, pathological fractures, neuropathic arthropathies, mental illness, significant surgical contraindications, osteoporosis, patients refusing to sign informed consent, and patients with signs of infection or degenerative OA. Primitive intervention varied between conservative measure by casting (three patients), application of ring fixation (seven patients), and open reduction and internal fixation (by plating in four patients and only screws in one patient). Two cases had infection following the primitive surgery which had been eradicated completely (evidenced by normalization of ESR, CRP, and total leukocytic count before implantation). Five cases had frontal laxity >15° while four cases had stiffness upon flexion. Full clinical examination is achieved to evaluate deformity both at rest and during weight-bearing, range of motions, ligament balancing, previous scarring, swelling, neurovascular status, and knee stability. There were ten patients with stable painful knee and five patients with unstable painful knee. Instability was due to ligamentous (real instability), bony (pseudo-instability), or combined (Table 1).

**Table 1:** Distribution of patients with post traumatic OA knee

	No.	%
Group 1: Post-traumatic stable painful knee	10	66.7
Group 2: Post-traumatic unstable painful knee (ligamentous, bony, or combined)	5	33.3
Total	15	100

Full laboratory investigations including complete blood cell count (CBC), erythrocyte sedimentation rate (ESR), C-reactive protein (CRP), and liver and kidney functions had been done.

**Fig. 1:**
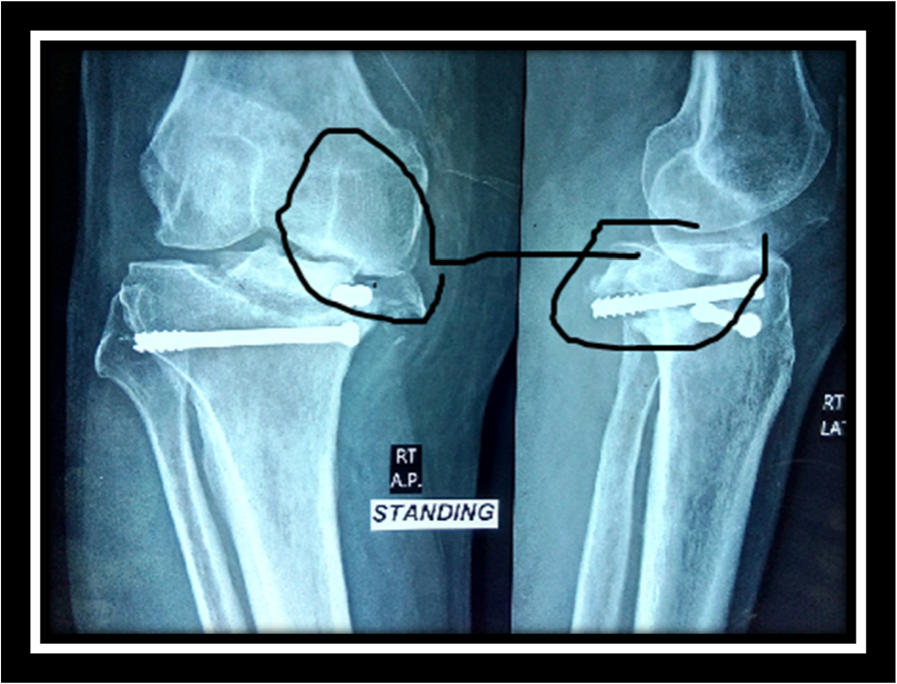
Intra-articular chondro-osseous defects

**Fig. 2:**
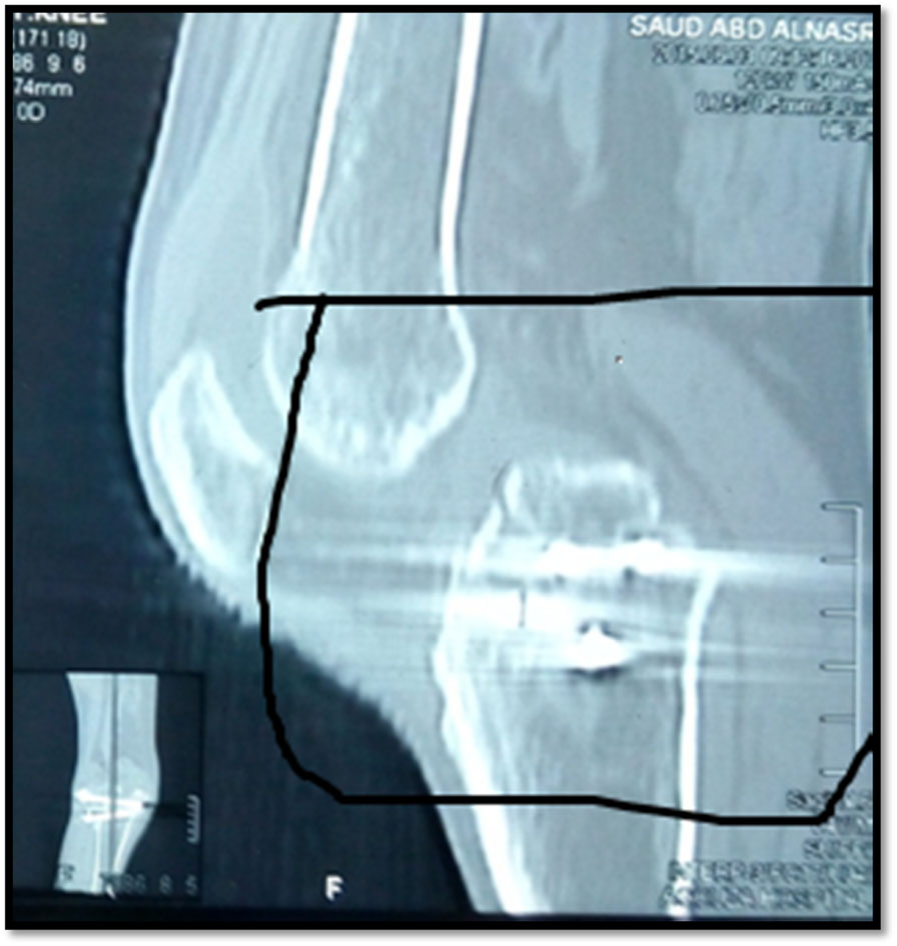
CT scan of posttraumatic unstable knee

Preoperative plain imaging studies (Fig. 1) were done to assess the fracture pattern, intra-articular chondro-osseous defects, limb axis, and deformity whether extra-articular or intra-articular through antero-posterior, lateral, and patellar axial view long-standing weight-bearing radiographs. Preoperative computed tomography scans were obtained for all cases to assess bone quality and possible defects (Fig. 2).

### Surgical procedure

Following preoperative clinical and radiological evaluation, approach, limb axis, degree of bone loss, choice of prosthesis, and level of constraint were planned. Low molecular weight heparin (Enoxaparine) given 12 h before surgery in a dose of 40 mg SC and started again 12 h postoperative and then given daily until the patient was discharged from the hospital and continued for 35 days.

Preoperative IV antibiotic (1 g third-generation cephalosporin) was administered to all patients. Two units of whole fresh blood to be ready for use according to intraoperative blood loss and preoperative patient hemoglobin level was prepared.

Epidural anesthesia was used in nine cases, spinal anesthesia in four patients, and general anesthesia in two patients. Pneumatic tourniquet was used in 11 cases and was deflated by the end of the procedure to perform meticulous hemostasis before wound closure.

Hip joint center was marked by a prominent mark (airway) over the palpable pulsations of the femoral artery 1 in below the mid-inguinal point (to guide the intraoperative check of limb alignment by alignment rods).

The limb washed with betadine solution and draped in a sterile towels.

**Fig. 3:**
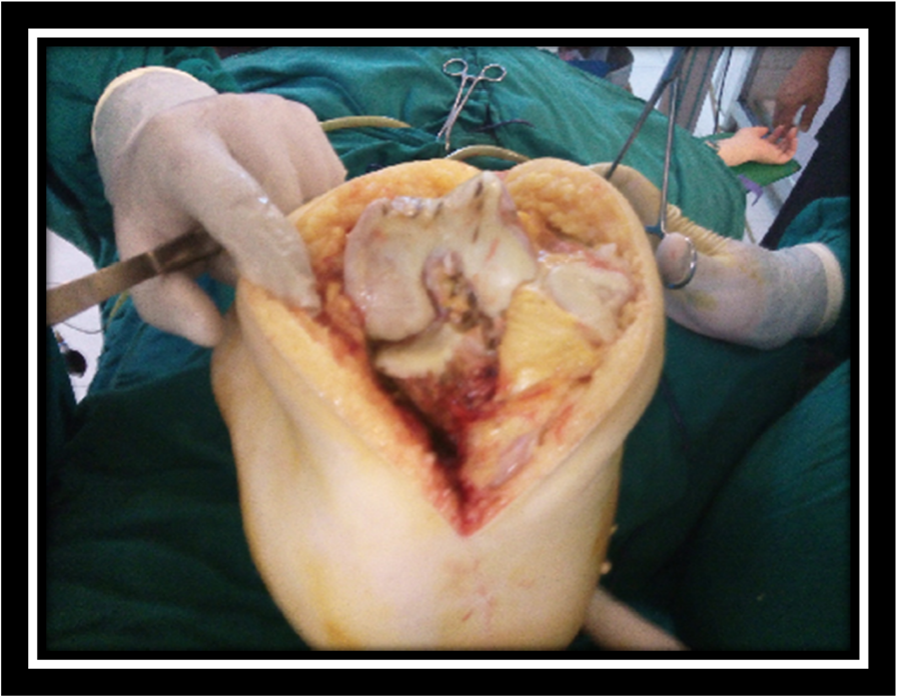
Medial parapatellar approach

A well-planned midline incision was utilized in ten cases and lazy S-shaped incision used in five cases (Fig. 3). We tried to involve the old scar within the new one. Fixation implant had been removed at the time of arthroplasty, and in one case, screws were removed through skin snips with lateral plate left without removal. Limited quadriceps release was performed in three patients and lateral retinacular release was performed in another two cases.

Five patients with post-traumatic varus deformity required medial release while one patient with valgus deformity required lateral release.

Extra-medullary guides was used in eight cases owing to distorted landmarks of upper tibia for proper tibial component orientation while meticulous reference using intramedullary guide was used in seven cases. The tibial cutting guide (left or right) was fitted over the extra-medullary rod.

Seven (out of ten patients) with painful stable arthritic knees had no tibial defect after initial resection of the tibia so they did not need any augmentation. The other three patients had grade I defect (according to Anderson Orthopedic Research Institute classification) [1] which was augmented by autologous impaction bone graft in two patients and cement augmentation in one patient. All these patients were treated with posterior stabilized (PS) prosthesis (Insall-Burstein II; Zimmer).

Of those with unstable painful knee patients (five patients), three had ligamentous instability and they were managed by constrained condylar knee prosthesis (Zimmer, Warsaw, IN).

**Fig. 4:**
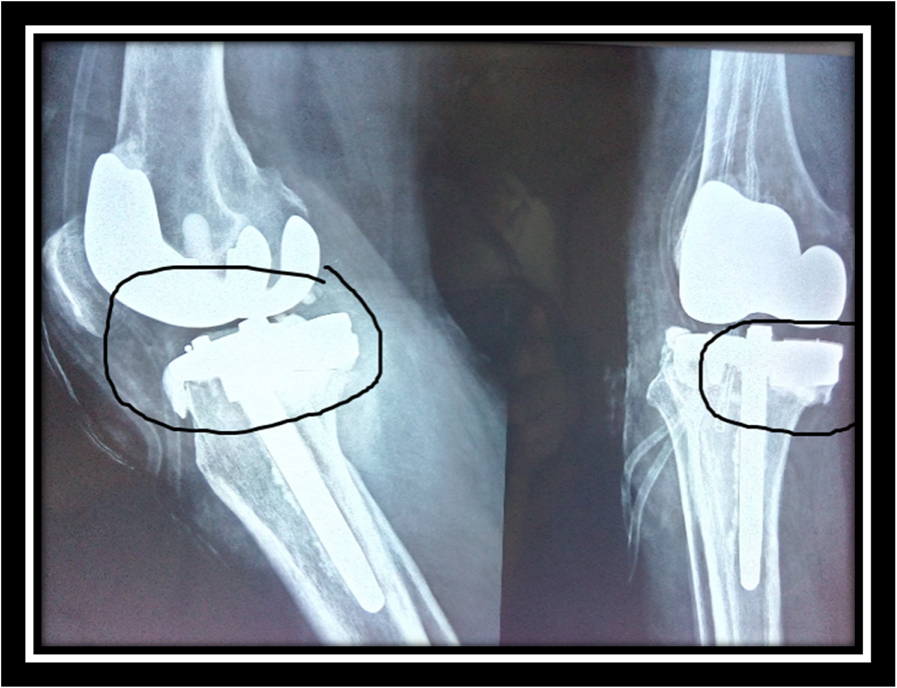
Stemmed constrained condylar knee prosthesis

The other two patients had osseous deficiency grade IIA in which metal augmentation was used together with stemmed constrained condylar knee prosthesis (CCK) implant with CCK liner to increase the level of constraint (Zimmer, Warsaw, IN) (Fig. 4). The distribution of the cases is shown in Table 2.

**Table 2:** Distribution of the cases according to the chosen implant

No. of cases		Description	Augmentation	Implant
10 cases	7 cases	Stable knee with no defect	No augmentation	PSA
	2 cases	Grade (I) defect	Impaction bone graft	
	1 case	Grade (I) defect	Impaction bone cement	
5 cases	3cases	Ligament instability		CCK
	2 cases	Grade IIA osseous deficiency	Metal augment	Stemmed CCK

*PSA* posterior stabilized arthroplasty, *CCK* constrained condylar knee

To minimize the risk of infection, we used antibiotic impregnated bone cement for implants fixation in all patients. No patella replacements had been done, only shaving of pathological cartilage with denervation of the periphery of the patella and removal of osteophytes. After completion of surgery, the patient’s knee was immobilized in a Jones compressive bandage and a knee immobilizer immediately postoperatively.

### Postoperative care

Careful monitoring of the vital signs, urine volume, drains, and distal circulation. A thin pillow was applied below the ankle to avoid postoperative flexion deformity and for slight elevation of the limb to decrease edema.

First dressing was applied after 48–72 h according to the amount of blood drained. Antibiotic and anticoagulant medications were continued. Static quadriceps and hamstring strengthening exercises and straight leg raising from the first postoperative day were performed. Weight bearing started next day of the operation. Patient was trained to bear as much weight as he can bear with the aid of the walker for 3 weeks then using a cane for another 3 weeks then full weight bearing without any aid.

Patients were followed up clinically and radiologically at 6 weeks, 6 months, 1 year, and periodically every 2 years with average duration of 6 years (range, 4–10 years) follow-up.

Clinical assessment at follow-up included both the knee society clinical and functional rating systems.

The function score was based on the patient’s ability to walk and climb the stairs (each rated 50 points), with 20 points subtracted when walking aids are used.

Radiological assessment was done through standing anteroposterior and lateral radiographs as well as tangential radiographs of the patella. The radiographic scoring system of the Knee Society [7] was used to determine the overall alignment of the limb, the respective size, fit and positions of the prosthetic components relative to the bone, and the presence of radiolucent lines in zones adjacent to the cement mantle. Femoral and tibial component alignment was assessed radiologically according to the Knee Society score with special emphasis on four angles:

α angle: Angle between a line tangential to the femoral component at the joint line and the anatomical axis of the femur in the antero-posterior view

β angle: The angle between line tangential to the tibial tray and the anatomical axis of the tibia in the antero-posterior view

σ angle (the tibial posterior slope angle): The angle between line tangential to the tibial tray and the anatomical axis of the tibia in the lateral view

**Fig. 5:**
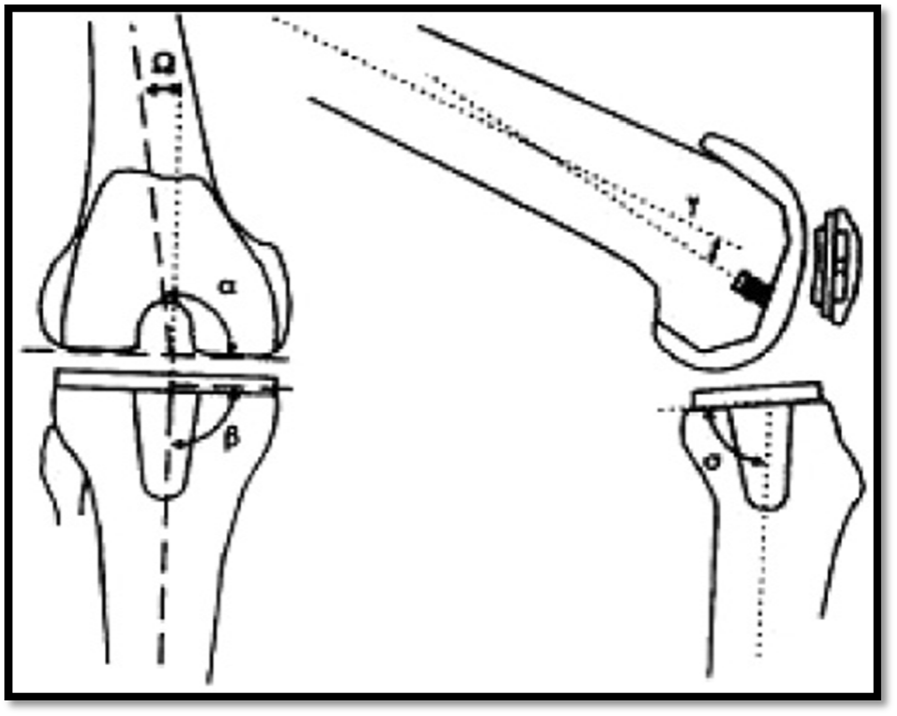
Schematic representation of aforementioned angles

γ angle: Between the anatomical axis of the femur and a line perpendicular to the center of the femoral component in the lateral view (Fig. 5)

**Fig. 6:**
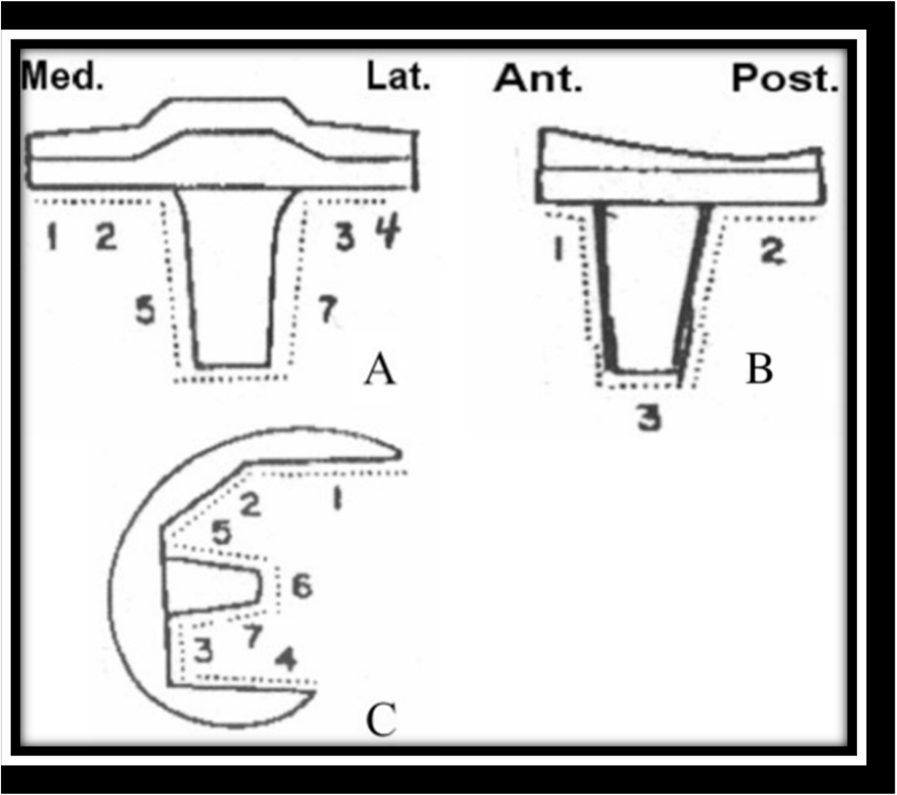
The Knee Society Roentgenographic Evaluation System. **a** Anteroposterior view of representative tibial component. **b** Lateral view of representative tibial component. **c** Lateral view of representative femoral component

Also, changes in thickness of polyethylene or asymmetry between the heights of the lateral and medial spaces on the anteroposterior radiograph made with the patient bearing weight were considered as possible evidence of polyethylene wear. The radiographs were evaluated for changes in the bone that were consistent with osteolysis and for progression and location of radiolucent lines. The presence or absence of radiolucent lines was evaluated with the system of the Knee Society, which involves seven tibial and seven femoral zones (Fig. 6).

Diagnosis of loosening of a component was made on the basis of three radiographic criteria: (1) a global radiolucent line, more than 1 mm thick, about the entire bone-cement interface; (2) a progressing radiolucent line, more than 1 mm thick, in any one zone; or (3) gross subsidence or shift of the component.

### Statistical analysis

Descriptive data were expressed as frequency and percentages and means with SD. A value of *p* < 0.05 was considered statistically significant. Statistical analyses were performed using SPSS 20.0.

## Results

This prospective case series study was conducted to evaluate the outcomes of total knee arthroplasty following post-traumatic arthritis in 15 patients (12 males). According to the clinical knee society scoring system, the mean pain score of the overall series at the last follow-up had significantly improved from 19 ± 9 preoperatively to 47 ± 16 (*p* < 0.001) (mean gain 28 points). The mean preoperative clinical knee society score (CKSS) was 43.6 ± 11.66 points which was increased to an average of 77.3 ± 13.88 points at the latest follow-up (mean gain 34 points). According to the knee society clinical scoring system, 11 patients (73.3%) were assessed as excellent, 2 patients (13.3 %) good, 1 patient (6.7%) fair, and 1 patient (6.7%) poor result (Table 3). The mean functional knee society score (FKSS) improved from 40 ± 16.3 points preoperatively to 76.6 ± 84 points at last follow-up (mean gain 36 points) and according to the knee society functional scoring system, 10 patients (66.6%) assessed as excellent, 3 patients (20%) good, 1 patients (6.7%) fair, and 1 patient (6.7%) poor results as shown in Table 4.

**Table 3:** Grading of knee clinical score

Knee clinical score	Frequency (%)
Excellent	11 patients (73.3)
Good	2 patients (13.3)
Fair	1 patients (6.7)
Poor	1 patients (6.7)
Total	15 patients (100)

**Table 4:** Grading of knee functional score

Knee functional score	Frequency (%)
Excellent	10 patients (66.6)
Good	3 patients (20)
Fair	1 patients (6.7)
Poor	1 patients (6.7)
Total	15 patients (100)

The mean clinical knee society score (CKSS) was improved in stable knee patients from 48.5 ± 16 points preoperatively to 81 ± 11 points in the last follow-up, while the unstable knee patients improved from 31.6 ± 14 points preoperatively to 70.7 ± 12 points at the last follow-up. The mean functional knee society score (FKSS) was improved in stable knee patients from 45.22 ± 13 points preoperatively to 79.5 ± 16 points postoperatively while in the unstable knee group, it was improved from 30.6 ± 14 points preoperatively to 72.7 ± 13 points at the last follow-up (Table 5).

**Table 5:** Results of stable and unstable knee groups

	Society score	Preoperative	Postoperative
Stable knees (10 patients)	CKSS	48.5 ± 16	81 ± 11
	FKSS	45.22 ± 13	79.5 ± 16
Unstable knees (5 patients)	CKSS	31.6 ± 14	70.7 ± 12
	FKSS	30.6 ± 14	72.7 ± 13

*CKSS* clinical knee society score, *FKSS* functional knee society score

Association between knee functional score and knee clinical score was documented preoperatively and at the last follow-up. Out of 11 patients who had excellent knee clinical score, 10 patients (90.9%) had excellent knee functional scores while 1 patient (9.1%) had good knee functional scores. The 2 patients who had good knee clinical score also had good knee functional score. One patient had fair score for both clinical and functional scores owing to development of infection. Another 1 patient had poor result for both clinical and functional scores owing to development of aseptic loosening which required revision (Table 6).

**Table 6:** Association between knee functional score and knee clinical score

Knee clinical score (KCSS)	Knee functional score (KFSS)				Total
	Excellent	Good	Fair	Poor	
Excellent	10	1	–	–	11
Good	–	2	–	–	2
Fair	–	–	1	–	1
Poor	–	–	–	1	1
Total	10	3	1	1	15

Statistical analyses were performed using SPSS 20.0 and the association between functional and clinical knee scores with Spearman rank correlation coefficient. The value of spearman was 0.319 preoperatively reaching 0.658 (*p* < 0.05). This signifies that there is a significant association between the knee clinical and functional scores.

## Complications

There were three cases (20%) in our study with complications, one patient was complicated by wound dehiscence and prolonged drainage for more than 7 days and was treated with oral antibiotics and daily sterile dressings until the wound closed completely. Another case was complicated by infection and improved by serial debridement. The third case had aseptic loosening and required revision surgery.

## Discussion

Post-traumatic arthritis (PTA) is defined as a structural damage following an injury to a joint minor or major, repetitive or single. It commonly affects more active individuals as they are more likely to participate in such activities that may cause injury (i.e., sports, blunt trauma, motor vehicle accidents) and it is often more notable in weight-bearing joints [9]. From radiological and pathological point of view, it is similar to primary osteoarthritis and it is estimated that 12% of all symptomatic osteoarthritis (OA) of the hip, knee, and ankle are due to post-traumatic arthritis PTA with a varied prevalence of 21–44% reported in the literature [13]. These injuries are usually intra-articular or extraarticular fractures of distal femur, tibial plateau, or complete dislocation of the knee, which often have required operative treatment [3]. The end result can be an arthritic knee with problems involving joint instability, stiffness, bony defects, malalignment secondary to intra- or extraarticular deformity, surgical scars, soft tissue abnormalities, and retained internal fixation devices. The lack of knowledge pertaining to the incidence of post-traumatic arthritis is due partly to the limited availability of long-term follow-up studies. The lack of control groups, variations in fracture classification and treatment methods, and different diagnostic criteria for the development of significant arthritis also make comparison of reports difficult [16].

Conservative treatment of post-traumatic knee OA varies from a combination of nonpharmacological and pharmacological modalities aiming at modifying the risk factors related to the pathogenesis. Activity modification, anti-inflammatory medications, ambulatory assist devices, and physical therapy are the mainstay of nonoperative treatment [6].

Other alternative therapies such as acupuncture, traditional Chinese medicine, and transcutaneous nerve stimulation have been implemented for managing PTA but they were not widely accepted due to insufficient evidence. However, conservative measures have major limitations, most notably a failure to successfully correct the underlying pathology, namely, abnormal joint loading resulting in continued disease progression. Despite marginal symptom relief with some nonsurgical therapies, none have been shown to alter disease progression [6].

A variety of surgical interventions had been described including (A) arthroscopic debridement and microfracture and this is indicated among patients with mild PTA with osteochondral lesions less than 15 mm in diameter and loading activities are permitted after 3 months [15]. (B) Osteochondral autograft or allograft transplantation. It has the advantage of transferring viable chondrocytes with optimal matching of graft and lesion to allow a stable bone-to-bone healing process [5]. However, this procedure necessitates prolonged period of nonweight-bearing and has been shown to have high failure and reoperation rates, particularly among older, less active patients [20]. (C) Joint distraction arthroplasty though controversial is indicated in highly motivated candidates with refractory pain, appropriate joint alignment, and preservation of motion (> 20°) who do not want to proceed with either joint arthrodesis or total joint arthroplasty [22]. Major drawback of this technique is the need for stringent follow-up and meticulous postoperative regimens [21]. (D) High tibial osteotomies are performed to realign the mechanical axis and thus redistribute the joint loading force in the knee, with the goal of delaying or stopping the degenerative cascade. However, some shortcomings as scarring of upper tibia and presence of internal fixation implant may disturb the future arthroplasty if needed [11]. (E) Arthrodesis. Being a salvage operation, it has limited indications owing to numerous drawbacks that evolved including limitation of daily patient activities.

Nowadays, total knee arthroplasty becomes a standard treatment for post-traumatic arthritic knee in terms of pain relief as well as knee stabilization with an appropriate range of motions and substantial functional improvement and an improvement in the quality of life of patients [13]. However, evidence on outcomes is sparse and there is paucity in the literature regarding the outcome of TKA performed for the treatment of PTA [17, 21]. Also, implanting knee prosthesis in such cases involves realigning the lower limb to ensure that the fixation of the implant components remains stable and minimizes long-term insert wear.

Different scoring systems had been utilized by several studies to judge the functional outcomes of TKA in PTA patients. The most valid and widely accepted is Knee Society Scoring criteria, which is composed of a functional and clinical knee scores. The functional score includes a patient assessment of walking distance, ability to climb stairs, and need for assistive devices, while the clinical knee score incorporates patient reported pain, ROM, alignment, and stability [10].

Literature reports show poorer results with TKA in post-traumatic arthritis than with primary arthritis. However, such studies [17] are few and involve a very limited number of patients. This is at odds with recent studies which report no difference in outcome scores between TKA after a fracture of the tibial plateau and TKA for primary osteoarthritis [11, 18].

Other studies reported an improvement in the functional and clinical knee scores of patients following TKA for PTA. Lizaur-Utrilla et al. [12] further reported that there were no significant differences in knee or pain scores of patients treated with TKA for PTA vs primary OA. Lunebourg et al. [15] while reporting significant improvement in scores noted lower postoperative scores for patients with PTA vs primary OA.

Collectively; these results indicate that TKA is an effective treatment for patients with PTA with functional improvement, as well as increased range of motion and reduction in pain. With regard to the lower postoperative scores noted above, despite significant improvement, it is reasonable to infer that this difference compared to patients undergoing TKA for primary OA may be due to differences in the preoperative status of the patients [12]. Thus, the postoperative difference observed in patients with PTA vs primary OA is not due to the intrinsic success of the procedure itself, but rather the poorer preoperative status of patients with PTA [17].

In our study, we presented a series of 15 post-traumatic knees in 15 patients, at a mean follow-up of 6 years, clinical KSS improved from a preoperative mean of 40.6 points to 77.3 points; the functional KSS improved from a preoperative mean of 40 to 76.6 points at follow-up.

We also present a classification not mentioned before in various studies regarding the choice of the prosthesis. The patients in our study were divided into two groups: stable painful arthritic knee group and unstable painful arthritic knee group. The results of patients with stable painful arthritic knee were comparable to those of unstable painful arthritic knee; we showed that patients with stable painful arthritic knee had higher mean values, but the preoperative state of those with unstable painful arthritic knee was poorer.

Lonner et al. [13] studied 30 patients with post-traumatic knee arthritis after intra-articular or metaphyseal fractures. At a mean follow-up of 46 months, an improvement of Knee Society Score (from a preoperative mean of 36 points to a postoperative mean of 78 points) was reported. Knee scores were considered excellent or good in 71% of cases.

The complication rate in our study was 26.7. In Lonner et al.’s study [13], there were complications in 57% of cases (aseptic failure 26%, septic failure 10%, and other complications 20%). Tibial loosening occurred mainly in cases where no stems were used or in cases with cementless tibial components. The authors therefore recommended the use of stems in cases of compromised metaphyseal bone stock.

Weiss et al. [21] in their study stated that the most common problems in patients with previous tibial plateau fracture were related to soft tissue healing, postoperative stiffness, and intraoperative extensor mechanism disruption. The authors showed that in patients for whom the goal is ideal limb and implant alignment, the results were similar to those of routine primary TKA. They also identified the single most important factor influencing outcome to be initial fracture treatment (correct incision, minimal periosteal stripping, anatomical reduction, use of bone graft).

They suggested that the outcome of TKA may be improved further by making special efforts to restore limb alignment, to ensure correct component positioning, and to manage soft tissue balance [19].

The limitations of our study include its small sample size. This is a consequence of the rarity of TKA after fractures around the knee. It is however, the first study (to our knowledge) classifying the patients into stable post-traumatic arthritic knee and unstable post-traumatic arthritic knee.

### Limitations of the study

Our study presents some limitations, namely small sample size owing to the rarity of TKA after fractures around the knee. Also, follow-up period is somewhat relatively short and lacking of comparative study. The technique itself has some limitations that it is contraindicated in cases associated with neurological deficits. It is, however, one of the little studies that classify the patients into stable post-traumatic arthritic knee and unstable post-traumatic arthritic knee with subsequent impact on choosing the prosthesis.

## Conclusion

Post-traumatic knee arthritis is particularly demanding for the surgeon and could be considered as a revision knee surgery. Every case needs good clinical and radiological evaluation. Planning is essential as latent infection (especially in cases of device in situ) must be excluded. Choosing the type of the prosthesis is a cornerstone in treating such cases and must be decided upon according to bone defect and ligament competence whether stable or unstable knee. The operation is difficult with higher rate of possible complications compared with routine primary total knee arthroplasty, majority of which occur in the perioperative period. Scarring from the initial trauma and prior surgeries, as well as the inherent technical difficulty of the operation, likely contribute to this complication rate. The technical challenges encountered can require skills, prosthetic systems, and methods usually reserved for complex revision arthroplasty.

## Abbreviations

CCK: Constrained condylar knee
CKSS: Clinical knee society score
FKSS: Functional knee society score
Knee OA: Knee osteoarthritis
Mean SD: Mean standard deviation
PS: Posterior stabilized
PTA: Post-traumatic arthritis
ROM: Range of motion
SC: Subcutaneous
vs: Versus

## References

[1] Anderson DD, Marsh JL, Brown TD (2011). The pathomechanical etiology of post-traumatic osteoarthritis following intraarticular fractures. Iowa Orthop J..

[2] Benazzo F, Rossi SM, Ghiara M (2014). Total knee replacement in acute and chronic traumatic events. Injury.

[3] Kester BS, Minhas SV, Vigdorchik JM, Schwarzkopf R (2016). Total knee arthroplasty for posttraumatic osteoarthritis: is it time for a new classification?. J Arthroplasty.

[4] Brown TD, Johnston RC, Saltzman CL, Marsh JL, Buckwalter JA (2006). Posttraumatic osteoarthritis: a first estimate of incidence, prevalence, and burden of disease. J Orthop Trauma..

[5] Buckwalter JA (1995). Osteoarthritis and articular cartilage use, disuse, and abuse: experimental studies. J Rheumatol Suppl.

[6] Crawford DC, Miller LE, Block JE (2013). Conservative management of symptomatic knee osteoarthritis: a flawed strategy?. Orthop Rev (Pavia)..

[7] Engh GA, Scuderi GR, Tria AJ (2006). Classification of bone defects femur and tibia. Knee arthroplasty handbook.

[8] Furman BD, Mangiapani DS, Zeitler E, Bailey KN, Horne PH, Huebner JL, Kraus VB, Guilak F, Olson SA (2014). Targeting proinflammatory cytokines following joint injury: acute intra-articular inhibition of interleukin-1 following knee injury prevents posttraumatic arthritis. Arthritis Res Ther..

[9] Insall JN, Dorr LD, Scott RD, Scott WN (1989). Rationale of the Knee Society clinical rating system. Clin Orthop Relat Res..

[10] Kadam RV, Yadav S, Chhallani A, Sharma C (2016). Prospective study of clinical and functional outcome of total knee replacement in osteoarthritic knee. Int J Res Orthop..

[11] Kanamiya T, Naito M, Hara M, Yoshimura I (2002). The influences of biomechanical factors on cartilage regeneration after high tibial osteotomy for knees with medial compartment osteoarthritis. Clin Arthrosc Obs Arthrosc..

[12] Lizaur-Utrilla A, Collados-Maestre I, Miralles-Muñoz FA, Lopez-Prats FA (2015). Total knee arthroplasty for osteoarthritis secondary to fracture of the tibial plateau. A prospective matched cohort study. J Arthroplasty.

[13] Lonner JH, Pedlow FX, Siliski JM (1999). Total knee arthroplasty for post-traumatic arthrosis. J Arthroplasty..

[14] Lotz MK, Kraus VB (2010). New developments in osteoarthritis. Posttraumatic osteoarthritis: pathogenesis and pharmacological treatment options. Arthritis Res Ther..

[15] Lunebourg A, Parratte S, Gay A, Ollivier M, Garcia-Parra K, Argenson JN (2015). Lower function, quality of life, and survival rate after total knee arthroplasty for posttraumatic arthritis than for primary arthritis. Acta Orthop..

[16] Osti L, Del Buono A, Maffulli N (2016). Arthroscopic debridement of the ankle for mild to moderate osteoarthritis: a midterm follow-up study in former professional soccer players. J Orthop Surg Res.

[17] Saleh KJ, Sherman P, Katkin P, Windsor R, Haas S, Laskin R, Sculco T (2001). Total knee arthroplasty after open reduction and internal fixation of fractures of the tibial plateau: a minimum five-year follow-up study. J Bone Joint Surg (Am)..

[18] Schenker ML, Mauck RL, Ahn J, Mehta S (2014). Pathogenesis and prevention of posttraumatic osteoarthritis after intra-articular fracture. J Am Acad Orthop Surg..

[19] Scott CEH, Davidson E, MacDonald DJ, White TO, Keatin JF (2015). Total knee arthroplasty following tibial plateau fracture: a matched cohort study. J Bone Joint Surg..

[20] VanTienderen RJ, Dunn JC, Kusnezov N, Orr JD (2017). Osteochondral allograft transfer for treatment of osteochondral lesions of the talus: a systematic review. Arthroscopy..

[21] Weiss NG, Parvizi J, Trousdale RT, Bryce RD, Lewallen DG (2003). Total knee arthroplasty in patients with a prior fracture of the tibial plateau. J Bone Joint Surg [Am]..

[22] Wiegant K, Van Roermund PM, Intema F (2013). Sustained clinical and structural benefit after joint distraction in the treatment of severe knee osteoarthritis. Osteoarthr Cartil..

